# Symmetric Dimethylarginine for Risk Stratification of Cardioembolic Stroke

**DOI:** 10.1007/s12975-026-01460-7

**Published:** 2026-07-14

**Authors:** Johanna Ernst, Ilai Mick Albert Darius Kaulbarsch, Svenja L. Jochmann, Maria M. Gabriel, Ramona Schuppner, Ralf Lichtinghagen, Jens Martens-Lobenhoffer, Hannelore Ehrenreich, Stefanie M. Bode-Böger, Anika Grosshennig, Karin Weissenborn, Rieke Ringlstetter, Gerrit M. Grosse

**Affiliations:** 1 Department of Neurology, Hannover Medical School, Carl-Neuberg Straße 1, Hannover, 30625 Germany; 2 KlinStrucMed Program, Dean´s Office for Academic Career Development, Hannover, Germany; 3 Department of Clinical Chemistry, Hannover Medical School, Hannover, Germany; 4 Institute of Clinical Pharmacology, Otto-von-Guericke University Magdeburg, Magdeburg, Germany; 5 Experimental Medicine, Central Institute of Mental Health, Medical Faculty Mannheim, Heidelberg University, Mannheim, Germany; 6 Institute of Biostatistics, Hannover Medical School, Hannover, Germany; 7 Department of Neurology, University Hospital Basel, Basel, Switzerland

**Keywords:** ADMA, Atrial fibrillation, Biomarkers, Cryptogenic stroke, Dimethylarginine, Ischemic stroke, L-arginine, SDMA

## Abstract

A significant proportion of ischemic strokes are classified as embolic stroke of undetermined source (ESUS), complicating secondary prevention. Nitric oxide metabolism is regulated by L-arginine (Arg), asymmetric (ADMA) and symmetric dimethylarginine (SDMA), contributing to increased cardiovascular risk. Previous studies suggest that SDMA and the ratio of Arg/SDMA help to detect atrial fibrillation (AF). We hypothesized that SDMA and Arg/SDMA improve diagnosis of cardioembolic stroke (CES), which may enable more accurate secondary prevention. This prospective nested case-control study included ischemic stroke patients (CES or ESUS) at Hannover Medical School between January 2022 and July 2023. Blood samples were collected after 24 h of stroke onset. ADMA, SDMA and Arg concentrations were measured and relevant biomarker ratios were calculated. Logistic regression and receiver operating curve analyses examined discriminatory biomarker performances for CES versus ESUS, alongside established clinical risk scores. 235 patients (107 CES, 128 ESUS) were analyzed. Arg/SDMA ratio and SDMA values showed comparable or better discriminatory power between CES and ESUS than clinical AF scores (AUC SDMA = 0.71 (95%CI: 0.64–0.77, *p* < 0.001); AUC Arg/SDMA = 0.74 (95%CI: 0.67–0.80, *p* < 0.001)). The same was true for AF detected after stroke (AFDAS) vs. ESUS. When combining Arg/SDMA-ratio and AS5F, the diagnostic accuracy was modestly improved compared to AS5F alone (DeltaAUC = 0.03 (95%CI: -0.06-0.11)). In conclusion, SDMA and Arg/SDMA ratio can serve as biomarkers for AF and AFDAS. While the additional predictive value in combination with clinical scores may be limited, these findings support the pathogenic role of the Arg/SDMA pathway in stroke pathophysiology.

**Trial Registration**: The study is retrospectively registered in the German Clinical Trial Register (StudyID DRKS00038459).

**Supplementary Information:**

The online version contains supplementary material available at https://doi.org/10.1007/s12975-026-01460-7.

## Introduction

Ischemic stroke is one of the most common causes of death and disability-adjusted life-year among non-communicable disorders globally [1]. Stroke has a high risk for recurrence which cumulates over time [2]. Therefore, optimizing secondary prevention measures is crucial.

Cryptogenic stroke comprises around 25% of all ischemic strokes, with corresponding challenges in assigning optimal secondary prevention [3]. In 2014, Hart et al. established the concept of embolic stroke of undetermined source (ESUS) [4] as a subgroup within cryptogenic stroke, assuming that a substantial proportion of these strokes are actually due to an underlying cardioembolic cause, most importantly atrial fibrillation (AF). Meanwhile, four randomized controlled trials were conducted to investigate a pragmatic approach comparing the effect of a general oral anticoagulation against acetylsalicylic acid as secondary prevention in patients with ESUS, but ultimately remained neutral [5–8]. Consequently, new diagnostic assessments need to be implemented to reduce the proportion of ESUS and determine the definite cause of stroke, such as subclinical AF. New biomarkers for risk stratification of cardioembolic mechanisms in ESUS might therefore play a crucial role in optimizing and personalizing secondary prevention [9–12].

Previous studies have shown that asymmetric dimethylarginine (ADMA) and symmetric dimethylarginine (SDMA), both endogenous methylated derivatives of the amino acid L-arginine (Arg), play distinct but interrelated pathophysiological roles in stroke. Arg serves as a substrate for the synthesis of nitric oxide (NO). ADMA inhibits the nitric oxide synthase, which leads to reduced bioavailability of NO [13]. High serum levels of ADMA were shown to be associated with atherosclerosis [14]. SDMA competes with Arg for cellular uptake, which indirectly reduces substrate availability for NO synthesis, contributing to endothelial dysfunction [15]. Elevated SDMA levels are associated with a higher cardiovascular risk and atrial cardiopathy including AF in the general population [16, 17]. In a pilot study, we have shown that SDMA as well as the ratio Arg/SDMA are potential biomarkers for identifying AF in patients hospitalized with embolic stroke. Therefore, both ADMA, SDMA and Arg not only play a role in stroke pathomechanisms but could also serve as biomarkers to support determining stroke etiology.

In this study, we aimed to replicate previous findings of the role of Arg and its derivatives in cardioembolic stroke and to investigate the potential incremental value of these biomarkers over established clinical risk scores to identify patients with cardioembolic origin of stroke.

## Methods

### Study Design and Ethics Approval

This is a prospective, monocentric case-control study embedded in a prospective observational cohort study (nested case-control study).

The corresponding ethics application was approved before the start of the study (Hannover Medical School ethics vote no. 9113_BO_S_2020). Each patient must have signed a written consent form if, after receiving thorough information, they agreed to participate in the study. If a patient is unable to give their consent, their legal representative was informed in accordance with the ethics decision and had to sign the consent form if, after receiving thorough information, they agreed to participate in the study.

This article was organized according to Strengthening the Reporting of Observational Studies in Epidemiology guidelines [18].

### Study Population

The inclusion criteria of the prospective observational cohort study were defined as an ischemic stroke confirmed by computed tomography or magnetic resonance imaging, availability of a blood sample and a signed informed consent form by the patient or proxy.

The stroke etiology was classified according to the TOAST criteria, that is large artery atherosclerosis, small vessel disease, cardioembolic stroke (CES), stroke of other determined etiology, or cryptogenic stroke [19]. ESUS, as a subgroup of cryptogenic strokes, was defined according to Hart [4], i.e. detection of an ischemic insult in cerebral imaging with CT or MRI and exclusion of lacunar infarcts, exclusion of relevant atherosclerosis of extra- or intracranial cerebral vessels with a lumen narrowing of more than 50%, exclusion of cardiac embolic causes by means of a 24-hour electrocardiogram (ECG) and transthoracic and/or transesophageal echocardiography and exclusion of other rare causes such as vasculitis, dissection, migraine or drug abuse. Classification of stroke etiology was done by two stroke experts (GMG, JE) according to the following procedure: JE has performed classification of stroke etiology in the first round. GMG has rated randomly selected 10% of the patients blinded to the first rating. In case of a lack of agreement a joint discussion took place to achieve agreement. The general portion of agreement from the first term rating after discussion was 94%. In case of a lack of agreement a joint discussion took place to achieve agreement.

To perform the here presented nested-case control study, we took a cohort including only ESUS and CES patients with a serum sample taken within 24 h after stroke onset as samples and controls.

From January 2022 to July 2023, 907 patients with ischemic stroke at Hannover Medical School were considered and consecutively included in the prospective observational cohort study. Of these, patients were excluded due to missing imaging evidence of stroke (*n* = 95), missing written consent (*n* = 109), missing blood samples in general (*n* = 54) and double inclusions (*n* = 3) as well as stroke etiology other than CES or ESUS (*n* = 251) and missing blood sample within the first 24 h after stroke onset (*n* = 160). (see Study flow chart: Fig. S-1, Supplementary information)

Therefore, a total of 235 patients were included in the nested case control study, 107 of them were classified as CES and 128 as ESUS.

Among the cardioembolic strokes, 60 patients had known atrial fibrillation (KAF) and 43 patients revealed AF detected after stroke during inpatient stay (AFDAS). 4 patients had a non-AF-related CES. Cardiac and ECG examinations were performed according to a standardized protocol as part of routine clinical practice. All patients received a standard ECG and systematic cardiac monitoring at our stroke unit for more than 24 h. Patients without known AF underwent an additional Holter-Monitoring for at least 24 h. In AFDAS patients AF was verified by stroke specialists and cardiologists.

### Data acquisition and eCRF

Every study participant was clinically phenotyped with high data granularity. The data were obtained from patients’ records, clinical routine, medical reports, study interviews and Picture Archiving and Communication System. Data collected included information on patients’ precondition, index stroke, medical and family history, stroke diagnostic in clinical routine, complications during hospitalization and information on discharge, as well as a structured interview by phone after 90 days.

All data collected were recorded in an electronic case report form (eCRF) using the online database system OpenClinica (OpenClinica, LLC, Needham, USA).

During their in-patient stay, patients underwent standardized stroke diagnostics due to clinical routine. This included duplex ultrasound examinations of intra- and extracranial vessels, transthoracic and/or transesophageal echocardiography, laboratory examinations, monitoring of vital signs and Holter ECG.

### Scores and Definition

AF was defined as supraventricular tachyarrhythmia for more than 30 seconds, characterized by uncoordinated atrial activation and ineffective atrial contraction [20]. Electrocardiographic characteristics include the absence of distinct P waves, irregular R-R intervals (when atrioventricular conduction is present) and irregular atrial activity.

Based on the available risk factors, we calculated the following AF risk scores for each patient: CHA₂DS₂-VASc [20] score (1 point each for congestive heart failure/left ventricular dysfunction, arterial hypertension, diabetes mellitus, vascular disease, age 65 to 74 years, female sex, and 2 points each for age ≥ 75 years and stroke), AS5F [21] score (Age: 0.76 points/year, Stroke Severity NIHSS ≤ 5 = 9 points, NIHSS > 5 = 21 points), HAVOC [22] score (1 point each for peripheral vascular disease and obesity (BMI ≥ 30 kg/m²), 2 points each for arterial hypertension, age ≥ 75 years, valve disease, coronary artery disease, and 4 points for congestive heart failure), Essen Stroke Risk Score (ESRS) [23] (1 point each for age 65 to 74 years, arterial hypertension, diabetes mellitus, previous myocardial infarction (MI), other cardiovascular disease except MI and AF, peripheral arterial disease, smoking, and 2 points for age ≥ 75 years).

### Laboratory Sampling

A venous blood sample was taken from each participating patient as part of the clinical routine within the first 24 h after stroke onset using residual samples. Blood was centrifuged for 15 min at 1.600 × g. Serum samples were stored until analysis at − 80 °C in the Hannover Unified Biobank (HUB) at Hannover Medical School.

### Biomarkers

Levels of ADMA, SDMA and Arg were measured using high-performance liquid chromatography-tandem mass spectrometry [24] at the Institute of Clinical Pharmacology at Magdeburg University.

In addition to the concentrations of the individual biomarkers, we also calculated respective ratios, i.e. Arg/ADMA, Arg/SDMA and ADMA/SDMA.

### Statistical Analysis

The statistical analysis and creation of figures were performed by using IBM SPSS Statistics (version 29.0.2.0, Chicago, USA) and SAS (version 9.4, Cary, USA). Group differences were detected using the Mann-Whitney U test for non-normally distributed data. For graphical depiction, boxplots with Tukey´s whiskers are displayed. Correlations between several vascular risk factors and biomarkers were calculated using the Spearman-rho test.

Univariate and multivariate binary logistic regression analysis was done to examine the association between the serum biomarkers as well as their ratios, adjusting for (1) the aforementioned AF-scores and (2) estimated glomerular filtration rate (eGFR) and smoking status.

Receiver operating characteristic (ROC) analysis, including the corresponding area under the curve (AUC), was conducted to analyze the ability of biomarkers and AF-scores alone and in combination with each other to discriminate between ESUS and CES reported for CES, unless indicated otherwise. The Youden-index was calculated to determine optimal cut-offs.

Biomarkers or ratios and clinical scores were included in a binary logistic regression model. The predicted probabilities derived from this model were utilized to perform a ROC analysis to evaluate the discriminative performance of the combined predictors.

A p-value of < 0.05 was considered statistically significant and can be interpreted as a signal for a difference.

## Results

Table 1 provides the demographic and clinical characteristics of the study cohort. Serum biomarkers were available for the entire cohort. Patients with CES were a decade older than in the ESUS group. ESUS patients had lower pre-morbid mRS values compared to CES. In-hospital-mortality was higher in CES patients. The NIHSS on admission was significantly higher in the CES group than in the ESUS group. Compared with ESUS patients, CES patients had a higher LAVI and had a lower eGFR.

**Table 1:** Epidemiological and clinical characteristics of the study collective

	CES (*n* = 107)	ESUS (*n* = 128)	*p*-value*
Male sex (*n*, (%))	54 (51)	69 (54)	0.599
Age in years (median, Q1-Q3)	81 (72–86)	71 (62–81)	< 0.001
BMI in kg/m² (median, Q1-Q3)	26 (24–30)	26 (24–29)	0.446
prior mRS (n, (%))			
0	57 (54.3)	90 (70.3)	-
1	2 (1.9)	7 (5.5)	-
2	10 (9.5)	8 (6.3)	-
3	17 (16.2)	12 (9.4)	-
4	18 (17.1)	11 (8.6)	-
5	1 (1)	0 (0)	0.028
mRS at discharge (n, (%))			
0	10 (9.3)	29 (22.7)	-
1	4 (3.7)	22 (17.2)	-
2	14 (13.1)	20 (15.6)	-
3	12 (11.2)	21 (16.4)	-
4	36 (33.6)	25 (19.5)	-
5	15 (14.0)	9 (7.0)	-
6	16 (15.0)	2 (1.6)	< 0.001
Residential condition before admission (n, (%))			
self-care	77 (73.3)	112 (87.5)	-
care at home	19 (18.1)	12 (9.4)	-
nursing home	9 (8.6)	4 (3.1)	0.017
NIHSS on admission (median, Q1-Q3)	10 (3–17)	3 (1–8)	< 0.001
NIHSS on discharge date (median, Q1-Q3)	3 (1–7)	1 (0–3)	< 0.001
LAVI (n, (%))			
normal (< 29 ml/m²)	2 (2.8)	35 (30.4)	-
increased (29–33 ml/m²)	3 (4.2)	10 (8.7)	-
moderate (34–39 ml/m²)	9 (12.7)	19 (16.5)	-
severe (> 39 ml/m²)	46 (64.8)	32 (27.8)	< 0.001
LVEF (n, (%))			
normal	50 (69.4)	99 (86.8)	-
mildly abnormal	9 (12.5)	9 (7.9)	-
moderately abnormal	9 (12.5)	2 (1.8)	-
severely abnormal	4 (5.6)	4 (3.5)	< 0.001
eGFR in ml/min (median, Q1-Q3)	63 (48–82)	75 (62–88)	0.002
Creatinine in mg/dl (median, Q1-Q3)	0.97 (0.80–1.28)	0.94 (0.78–1.08)	0.128
Consuming alcohol (n, (%))			
never	65 (61.9)	71 (57.3)	-
1-5x/week	23 (21.9)	33 (26.6)	-
daily	16 (15.2)	20 (16.1)	-
undefined	1 (1)	0 (0)	0.789
Smoking status (n, (%))			
never smoked	60 (56.1)	49 (38.3)	-
current smoker	17 (15.9)	33 (25.8)	-
ex-smoker	28 (26.2)	43 (33.6)	0.051

*Based on Mann-Whitney U test for continuous variables and chi-square test for categorical variables

There were missing values for: prior mRS, residential condition before admission, NIHSS on admission (each < 1%), NIHSS on discharge date (2%), LAVI (34%), LVEF (21%), GFR (< 1%), Creatinine (< 1%), Consuming alcohol (3%), smoking status (4%)

Q1: first quartile, Q3: third quartile, BMI: body mass index, mRS: modified Rankin Scale, NIHSS: National Institutes of Health Stroke Scale, LAVI: left atrial volume index, LVEF: left ventricular ejection fraction, eGFR: estimated glomerular filtration rate

**Fig. 1:**
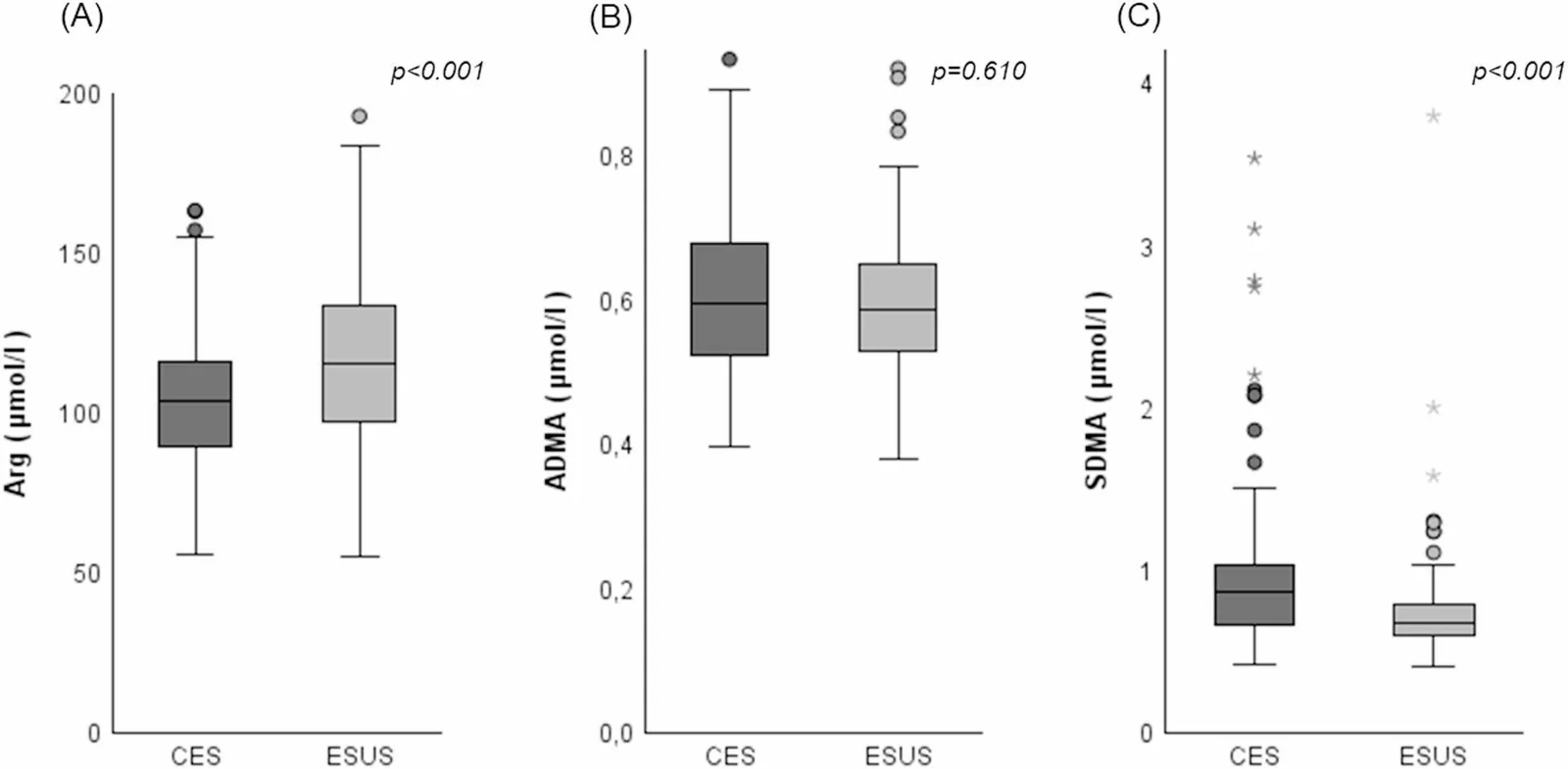
Comparison of serum concentrations of (**A**) L-arginine (Arg), (**B**) asymmetric dimethylarginine (ADMA) and (**C**) symmetric dimethylarginine (SDMA) between stroke etiologies

As shown in Fig. 1, serum levels of Arg were substantially lower in CES patients (median: 103.66 µmol/l, Q1-Q3: 89.42-116.06 µmol/l) compared to ESUS (median: 115.29 µmol/l, Q1-Q3: 97.32-133.55 µmol/l). SDMA levels were considerably higher in patients with AF (median: 0.86 µmol/l, Q1-Q3: 0.65–1.03 µmol/l) than in ESUS (median: 0.67 µmol/l, Q1-Q3: 0.59–0.79 µmol/l). There was no substantial difference regarding levels of ADMA in CES (median: 0.59 µmol/l, Q1-Q3: 0.52–0.68 µmol/l) and ESUS (median: 0.58 µmol/l, Q1-Q3: 0.52–0.64 µmol/l). Comparing biomarkers between AFDAS and ESUS patients revealed similar results as comparisons between CES and ESUS as shown in the Supplementary information (Fig. S-2).

**Fig. 2:**
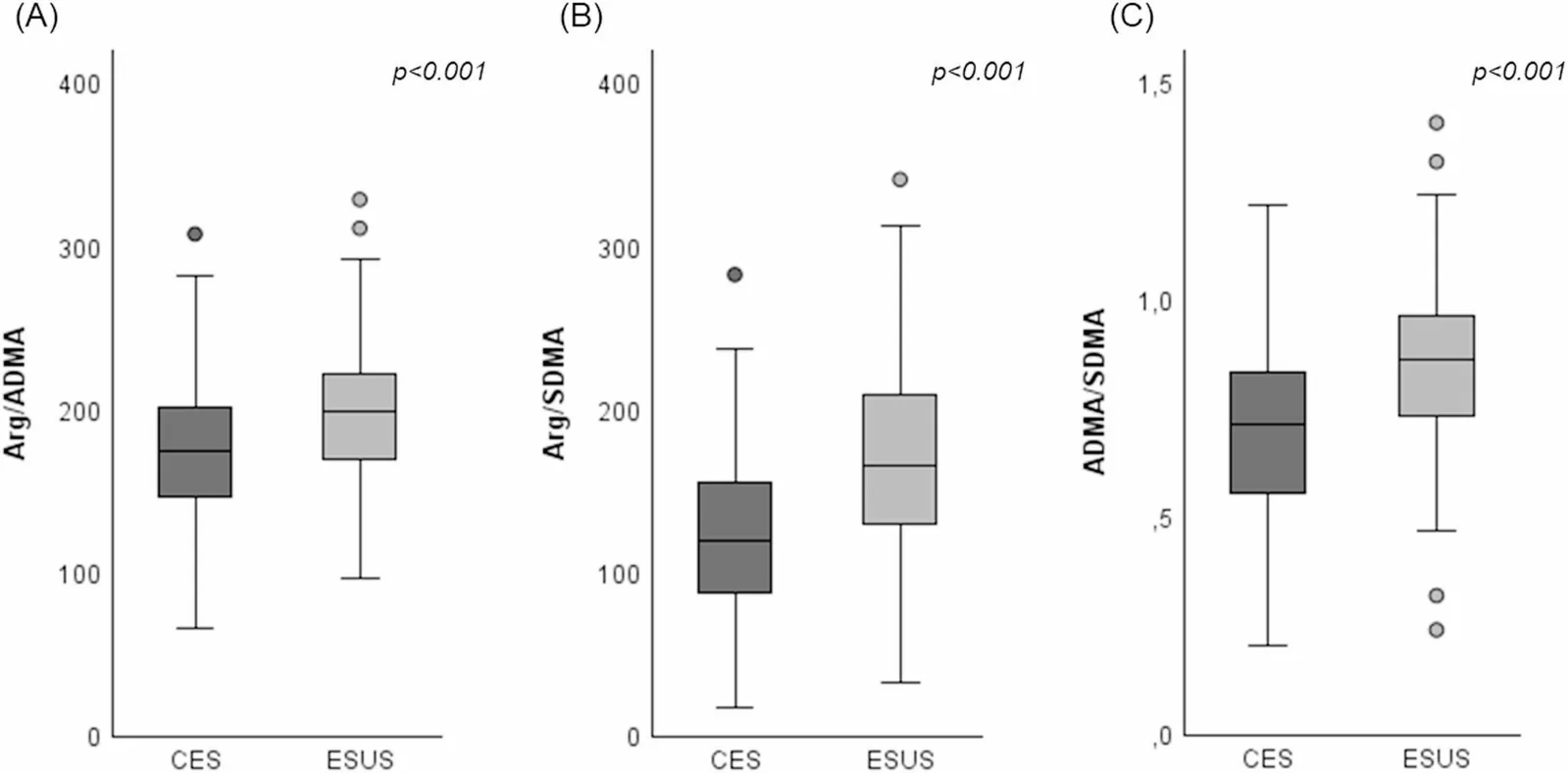
Comparison of biomarker (L-arginine (Arg), asymmetric dimethylarginine (ADMA), symmetric dimethylarginine (SDMA)) ratios between stroke etiologies: (**A**) Arg/ADMA, (**B**) Arg/SDMA and (**C**) ADMA/SDMA

The ratio of Arg/ADMA was significantly lower in patients with CES (median: 175.31, Q1-Q3: 145.71-202.27) compared to ESUS (median: 198.72, Q1-Q3: 170.31-223.52). Arg/SDMA was also lower in patients with AF (median: 119.64, Q1-Q3: 88.35-156.56) than with ESUS (median: 165.53, Q1-Q3: 130.41-209.25). ADMA/SDMA was substantially higher for ESUS (median: 0.86, Q1-Q3: 0.73–0.97) than for CES (median: 0.71, Q1-Q3: 0.55–0.84). (Fig. 2) Biomarker ratios comparing AFDAS and ESUS patients were provided in the Supplementary information (Fig. S-3).

ROC-analysis to distinguish between CES and ESUS revealed an AUC of 0.519 (95% CI: 0.44–0.60, *p* = 0.615) for ADMA, an AUC of 0.645 (95% CI: 0.58–0.72, *p* < 0.001) for Arg and an AUC of 0.708 (95% CI: 0.64–0.76, *p* < 0.001) for SDMA.

Equally well performing SDMA cut-offs to discriminate CES from ESUS were 0.766 µmol/l (sensitivity: 0.626, specificity: 0.734) as well as 0.803 µmol/l (sensitivity: 0.579, specificity: 0.781). For Arg the best performing cut-off to discriminate CES from ESUS was 113.374µmol/l (sensitivity: 0.555, specificity 0.710).

ROC for biomarker ratios revealed an AUC of 0.74 (95% CI: 0.67–0.8, *p* < 0.001) for the best performing biomarker ratio Arg/SDMA, an AUC of 0.66 (95% CI: 0.59–0.73, *p* < 0.001) for Arg/ADMA and an AUC of 0.72 (95% CI: 0.65–0.78, *p* < 0.001) for ADMA/SDMA. For Arg/SDMA, the ratio with the highest AUC, the best performing cut-off for discrimination between CES and ESUS was 138.036 (sensitivity: 0.626, specificity: 0.742).

**Fig. 3:**
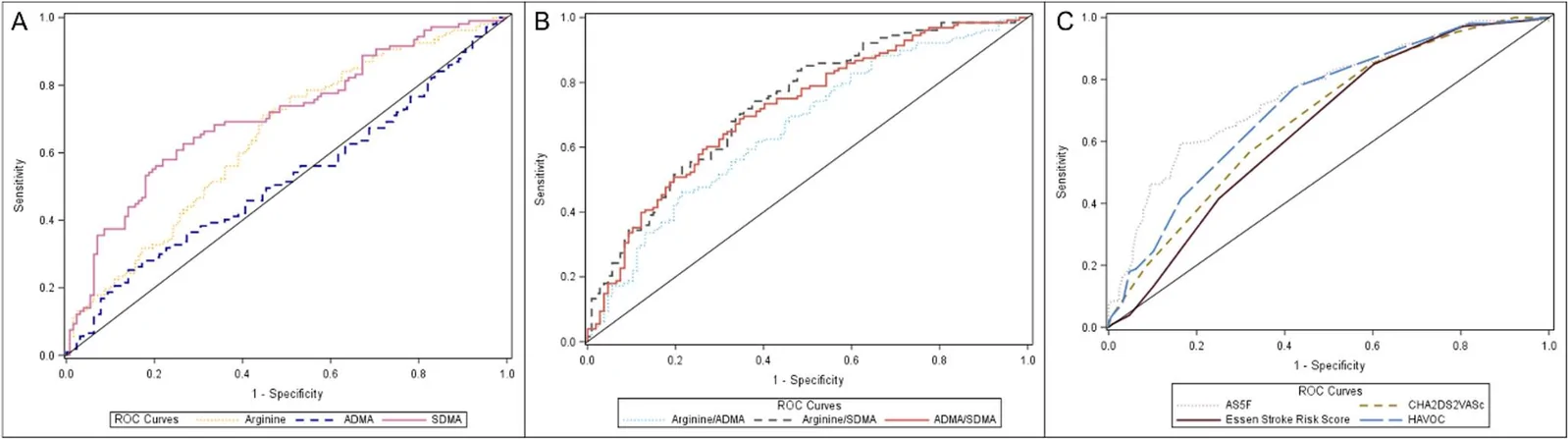
ROC-analysis for distinguishing between CES and ESUS with (**A**) biomarker levels, (**B**) biomarker ratios and (**C**) AF-scores

Among AF risk scores, the AS5F score had the highest AUC with 0.76 (95% CI: 0.64–0.82, *p* < 0.001), while the AUC for HAVOC was 0.72 (95% CI: 0.66.0.78, *p* < 0.001), for CHA₂DS₂-VASc 0.68 (95%CI: 0.61–0.74), *p* < 0.001) and for ESRS 0.65 (95%CI: 0.58–0.72, *p* < 0.001) (Fig. 3). Results for biomarker, biomarker-ratios and clinical scores comparing AFDAS and ESUS were similar (Fig. S-4).

**Fig. 4:**
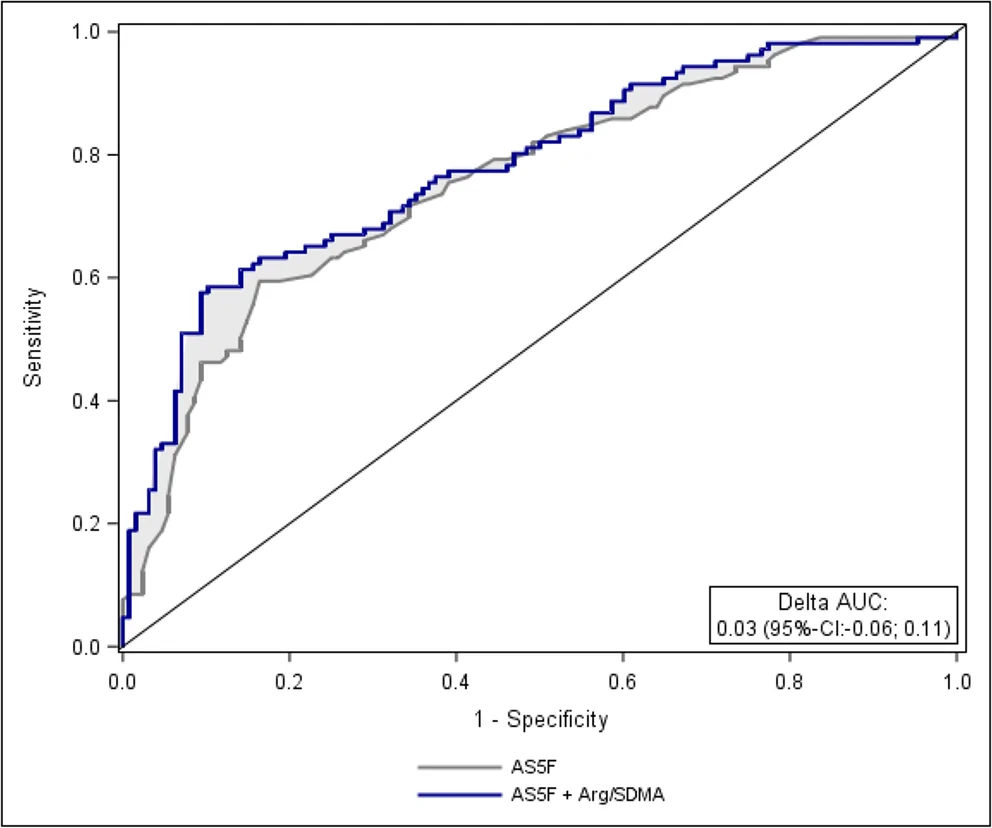
Delta-AUC comparing AS5F-score combined with Arg/SDMA ratio to isolated AS5F-score

When combining biomarker and clinical score to evaluate the discriminative performance of the combined predictors, the combination of AS5F and Arg/SDMA-ratio outperformed all other individual predictors or combinations with an AUC of 0.78 (95% CI: 0.73–0.84) (see Table 2 for further values). But to mention, that the additional value of Arg/SDMA added to AS5F-score is only modest compared with the isolated AS5F-score (Delta AUC = 0.03 (95%CI: -0.06-0.11)) (Fig. 4). Even, if comparing AS5F in combination with Arg/SDMA to the isolated clinical score for patients with AFDAS versus ESUS, the incremental value remained limited (Fig. S-5).

**Table 2:** Overview on diagnostic performance of biomarkers and clinical AF scores for CES

CES vs. ESUS							
	Clinical Stroke score	ADMA	SDMA	Arg	Arg/ADMA	Arg/SDMA	ADMA/SDMA
		OR per increase of 1 µmol/l = 2.58 (95%CI: 0.21–32.43) *p* = 0.463 AUC = 0.52 (95%CI: 0.44–0.60), *p* = 0.615 Optimal cut-off (Youden-Index): 0.680µmol/l, Sensitivity: 0.252, Specificity: 0.859	OR per increase of 1 µmol/l = 5.98 (95%CI: 2.23–16.07) *p* < 0.001 AUC = 0.71 (95%CI: 0.64–0.76), *p* < 0.001 Optimal cut-off (Youden-Index): 0.803 µmol/l, Sensitivity: 0.579, Specificity: 0.781 Youden-Index Value: 0.766 µmol/l, Sensitivity: 0.626, Specificity: 0.734	OR per increase of 1 µmol/l = 0.98 (95%CI: 0.97–0.99) *p* < 0.001 AUC = 0.65 (95%CI: 0.58–0.72), *p* < 0.001 Optimal cut-off (Youden-Index): 113,374 µmol/l, Sensitivity: 0.555, Specificity: 0.710	OR per increase of 1 = 0.986 (95%CI: 0.98–0.99) *p* < 0.001 AUC = 0.66 (95%CI: 0.59–0.73), *p* < 0.001 Optimal cut-off (Youden-Index): 143.415, Sensitivity: 0.785, Specificity: 0.461	OR per increase of 1 = 0.984 (95%CI: 0.98–0.99) *p* < 0.001 AUC = 0.74 (95%CI: 0.67–0.8), *p* < 0.001 Optimal cut-off (Youden-Index): 138.038, Sensitivity: 0.626, Specificity: 0.742	OR per increase of 1= 0.02 (95%CI: 0.004–0.08) *p* < 0.001 AUC = 0.72 (95%CI: 0.65–0.78), *p* < 0.001 Optimal cut-off (Youden-Index): 0.689, Sensitivity: 0.654, Specificity: 0.688
+AS5F	OR per point increase = 1.10 (95%CI: 1.06–1.13) *p* < 0.001 AUC = 0.76 (95%CI: 0.64–0.82), *p* < 0.001	OR = 1.91 (95%CI: 0.11–32.61) *p* = 0.653 AUC = 0.76 (95%CI: 0.7–0.82), *p* < 0.001	OR = 2.56 (95%CI: 1.09–6.03) *p* = 0.032 AUC = 0.77 (95%CI: 0.71–0.83), *p* < 0.001	OR = 0.98 (95%CI: 0.97 − 0.99) *p* = 0.041 AUC = 0.76 (95%CI: 0.70–0.82), *p* < 0.001	OR = 0.99 (95%CI: 0.985-1.00) *p* = 0.039 AUC = 0.77 (95%CI: 0.71–0.83), *p* < 0.001	OR = 0.99 (95%CI: 0.98-1.00) *p* < 0.001 AUC = 0.78 (95%CI: 0.72–0.84), *p* < 0.001	OR = 0.08 (95%CI: 0.02–0.4) *p* = 0.002 AUC = 0.78 (95%CI: 0.72–0.84), *p* < 0.001
+CHA₂DS₂-VASc	OR per point increase = 1.72 (95%CI: 1.38–2.16) *p* < 0.001 AUC = 0.68 (95%CI: 0.61–0.74), *p* < 0.001	OR = 1.26 (95%CI: 0.09–18.44) *p* = 0.868 AUC = 0.68 (95%CI: 0.61–0.75), *p* < 0.001	OR = 3.06 (95%CI: 1.18–7.88) *p* = 0.021 AUC = 0.72 (95%CI: 0.65–0.78), *p* = 0.001	OR = 0.98 (95%CI: 0.97–0.99) *p* = 0.002 AUC = 0.72 (95%CI: 0.65–0.78), *p* < 0.001	OR = 0.99 (95%CI: 0.98-1.00) *p* = 0.003 AUC = 0.72 (95%CI: 0.65–0.78), *p* < 0.001	OR = 0.987 (95%CI: 0.98–0.99) *p* < 0.001 AUC = 0.75 (95%CI: 0.69–0.81), *p* < 0.001	OR = 0.05 (95%CI: 0.01–0.22) *p* < 0.001 AUC = 0.74 (95%CI: 0.67–0.80), *p* < 0.001
+HAVOC	OR per point increase = 1.44 (95%CI: 1.25–1.65) *p* < 0.001 AUC = 0.72 (95%CI: 0.66–0.78), *p* < 0.001	OR = 1.25 (95%CI: 0.08–19.09) *p* = 0.871 AUC = 0.73 (95%CI: 0.66–0.79), *p* < 0.001	OR = 2.59 (95%CI: 1.02–6.56) *p* = 0.045 AUC = 0.74 (95%CI: 0.68–0.81), *p* = 0.001	OR = 0.98 (95%CI: 0.97–0.99) *p* = 0.004 AUC = 0.75 (95%CI: 0.68–0.81), *p* < 0.001	OR = 0.99 (95%CI: 0.98-1.00) *p* = 0.006 AUC = 0.75 (95%CI: 0.69–0.81), *p* < 0.001	OR = 0.988 (95%CI: 0.98–0.99) *p* < 0.001 AUC = 0.76 (95%CI: 0.7–0.82), *p* < 0.001	OR = 0.06 (95%CI: 0.01–0.30) *p* < 0.001 AUC = 0.75 (95%CI: 0.68–0.81), *p* < 0.001
+ESRS	OR per point increase = 1.48 (95%CI: 1.19–1.84) *p* < 0.001 AUC = 0.65 (95%CI: 0.58–0.72), *p* < 0.001	OR = 2.92 (95%CI: 0.21–39.99) *p* = 0.422 AUC = 0.65 (95%CI: 0.58–0.72), *p* < 0.001	OR = 4.12 (95%CI: 1.58–10.71) *p* = 0.004 AUC = 0.72 (95%CI: 0.65–0.78), *p* < 0.001	OR = 0.979 (95%CI: 0.97–0.99) *p* < 0.001 AUC = 0.71 (95%CI: 0.64–0.77), *p* < 0.001	OR = 0.987 (95%CI: 0.98–0.99) *p* < 0.001 AUC = 0.71 (95%CI: 0.65–0.79), *p* < 0.001	OR = 0.985 (95%CI: 0.98–0.99) *p* < 0.001 AUC = 0.75 (95%CI: 0.69–0.81), *p* < 0.001	OR = 0.03 (95%CI: 0.01–0.14) *p* < 0.001 AUC = 0.73 (95%CI: 0.66–0.79), *p* < 0.001

Binary logistic regression (reported for CES, unless indicated otherwise) was performed, reporting odds ratios (ORs) per 1 µmol/L increase with 95% confidence intervals (CI). The area under the ROC curve (AUC) with 95% CI was calculated. Optimal cut-offs were determined according to the Youden index, with corresponding sensitivity and specificity. Analyses were performed for biomarkers, biomarker ratios, clinical scores, and combined models

When adjusting biomarkers and biomarker ratios for eGFR and smoking status in binary logistic regression to detect CES, results of effect estimates are the following for ADMA (OR per increase of 1 µmol/l = 1.34, 95% CI: 0.09–20.35, *p* = 0.833), SDMA (OR per increase of 1 µmol/l = 5.44, 95% CI: 1.54–19.25, *p* = 0.009), Arg (OR per increase of 1 µmol/l = 0.98, 95% CI: 0.9–0.99, *p* = 0.002), Arg/ADMA (OR per increase of 1 = 0.99; 95% CI: 0.98–0.99, *p* = 0.003), Arg/SDMA (OR per increase of 1 = 0.98, 95% CI: 0.98–0.99, *p* < 0.0001) and ADMA/SDMA (OR per increase of 1 = 0.01, 95% CI: 0.001–0.07, *p* = < 0.0001).

**Fig. 5:**
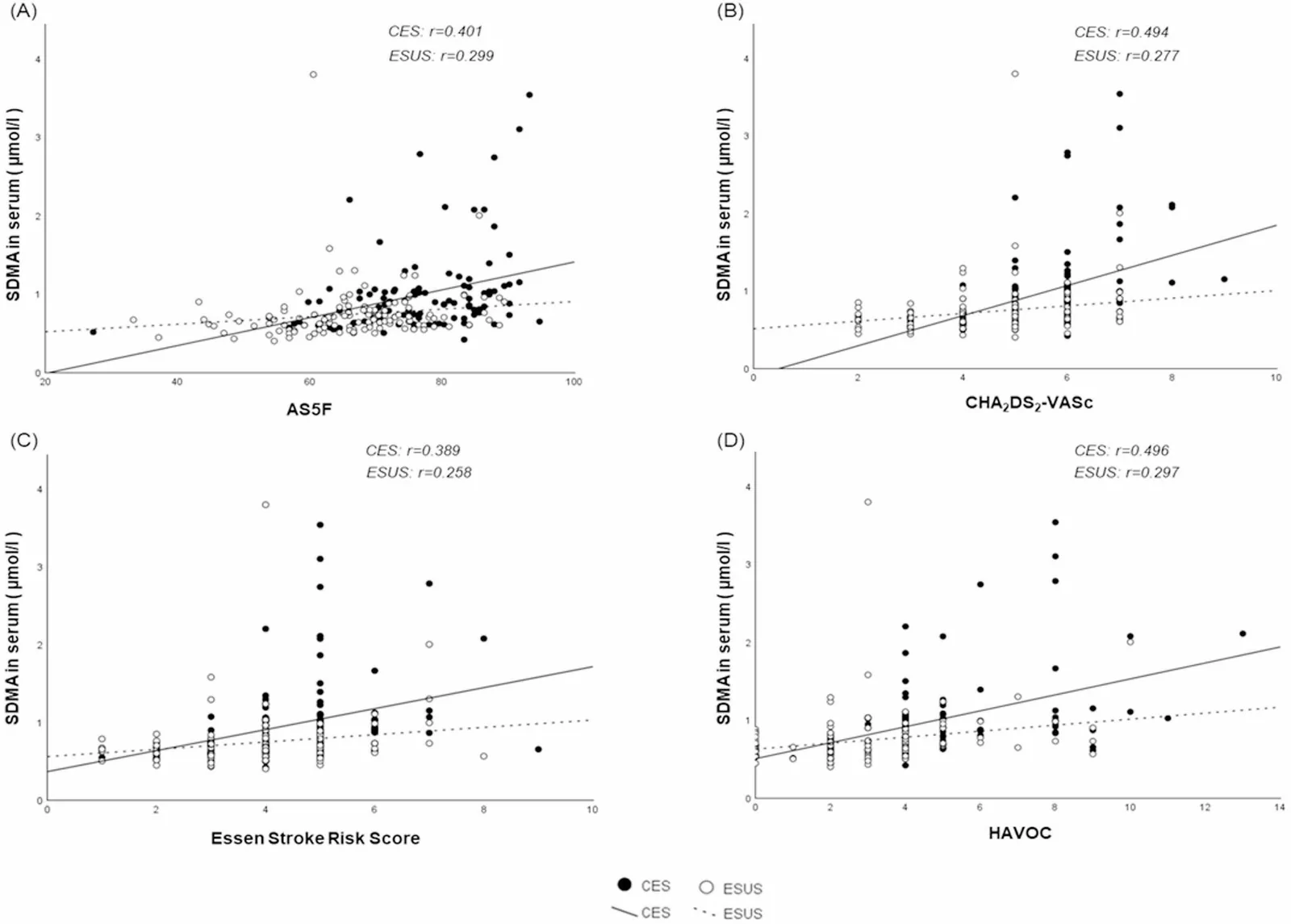
Correlation of SDMA with AF-scores in CES and ESUS: (**A**) AS5F, (**B**) CHA₂DS₂-VASc, (**C**) Essen Stroke Risk Score and (**D**) HAVOC

For correlations between serum SDMA levels and AF scores, stratified by either CES or ESUS, see Fig. 5. Positive correlations could be shown for all scores in both etiologies. Correlation coefficients were considerably lower in the ESUS group: AS5F (*r* = 0.299), CHA₂DS₂-VASc (*r* = 0.277), ESRS (*r* = 0.258), HAVOC (*r* = 0.297), as compared with CES: AS5F (*r* = 0.401), CHA₂DS₂-VASc (*r* = 0.494), ESRS (*r* = 0.389), HAVOC (*r* = 0.495).

## Discussion

Secondary stroke prevention fundamentally depends on identifying the underlying cause of the index event and risk factor burden. While patients with AF benefit markedly from anticoagulation, the optimal management of embolic stroke of undetermined source (ESUS) remains challenging [25, 26]. Multiple large randomized controlled trials have demonstrated that empiric anticoagulation is not beneficial for unselected ESUS populations, underlining the urgent need for improved risk stratification methods based on pathophysiological mechanisms rather than broad diagnostic categories [5–8]. Therefore, risk stratification of an underlying cardioembolic mechanism is still crucial but comes along with substantial limitations in clinical routine, such as labor- and resource-intensive nature of prolonged ECG monitoring [27]. Moreover, emerging evidence emphasizes that structural atrial remodeling, i.e. atrial cardiopathy, may confer thromboembolic risk independent of AF episodes [28]. Importantly, patients with AF detected after stroke (AFDAS) may benefit less from anticoagulation, underscoring that type and burden of left atrial disease is crucial in this regard [29, 30]. Therefore, identifying accessible and robust biomarkers reflecting this risk is paramount for precision medicine in stroke care.

Arg and its derivatives are biomarkers of endothelial (dys)function which is considered a cornerstone in the pathophysiology of atrial cardiopathy [31]. We previously investigated Arg, ADMA and SDMA in the course of a pilot study [12] in ESUS and AF-related strokes and were able to show that SDMA, as well as the Arg/SDMA ratio predict AF and are correlated with LAVI, an established measure of atrial cardiopathy [17]. In accordance, a recent investigation of the FIND-AF study found that baseline SDMA values are predictive for AF episodes in Holter monitoring following stroke [32].

Our current results, in a larger and independent cohort, confirm that SDMA concentrations and Arg/SDMA ratio distinguish CES from ESUS, performing as well as established AF risk scores. Only the AS5F-score achieved slightly better performance than the biomarkers alone. Even after adjusting for eGFR and smoking status, results were almost unchanged. However, combining biomarkers to clinical scores only modestly improved the discriminatory accuracy.

In accordance with previous studies, ADMA levels and related ratios did not show a significant discriminatory power, underlining the specific relevance of SDMA and Arg/SDMA in this context.

Recent studies underscored the predictive value of SDMA in the context of cardiovascular disorders. Emrich et al. [16] were able to demonstrate that elevated levels of SDMA in a cohort of chronic kidney disease patients were at increased risk for cardiovascular events (including stroke) during a 5-year follow-up period, even after adjusting for risk factors including renal function, age, total cholesterol and existing cardiovascular disease. In a sub-study of the ARISTOTLE (Apixaban for Reduction in Stroke and Other Thromboembolic Events in Atrial Fibrillation) trial in CES patients, SDMA levels correlated with the CHA₂DS₂-VASc score regarding the prediction of stroke events [33]. In accordance with these findings, we found significant correlation of SDMA with all considered AF-scores in the CES group, whereas correlation in the ESUS group was markedly lower.

In patients with ischemic stroke, Cordts et al. [34] reported an association of a low circulating homoarginine (hArg)/SDMA ratio with an increased risk for stroke according to the CHA₂DS₂-VASc-Score. Atzler et al. [35] were able to identify an independent inverse association of hArg levels with major adverse cardiac events and aortic wall thickness. This suggests that hArg may have a protective effect in early atherosclerosis as an underlying mechanism for cardiovascular events, such as stroke. Choe et al. [36] could show that stroke patients with high hArg/SDMA ratio experienced fewer adverse events compared with those with low hArg/SDMA ratios within 30 days after stroke. Furthermore, hArg/SDMA ratios inversely correlated with the NIHSS, as measure of stroke severity. Likewise, hArg/SDMA was also shown to be a prognostic marker of stroke in the long-term. In our study, we could also show lower levels of Arg and Arg/SDMA in the CES group, which had a higher NIHSS at admission and discharge and a higher mRS at admission. Of note, Hanff et al. [37] found that there is merely a borderline correlation between plasma levels of hArg and Arg in healthy patients. Therefore, the parallelism of the aforementioned results with our findings must be interpreted with caution.

In the FIND-AF trial [32] SDMA had an AUC of 0.676, consitent with our findings with an AUC of 0.653. SDMA plasma concentration > 0.571 µmol/l significantly predicted the presence of AF in ECG (sensitivity: 0.786, specificity: 0.572) in the FIND-AF trial. In our study, we were able to confirm a similar optimal cutoff for SDMA in terms of differentiating AFDAS from ESUS with 0.613 µmol/l. Of note, these results are also consistent with our previous work [17].

Beyond diagnostic and predictive purposes, the Arg/SDMA system could also have therapeutic implications for stroke prevention. E.g., in a double-blind, randomized, placebo-controlled crossover study, the daily oral intake of 125 mg L-hArg once daily for 4 weeks in a cohort of 20 volunteers increased the plasma levels of Arg by four to seven times over baseline [38]. It would be of interest to investigate how hArg treatment may constitute a novel approach for (secondary) stroke prevention and how its intake affects serum concentrations in patients with pre-existing cardiovascular disease. However, there is no evidence to date that higher concentrations of hArg or Arg cause lower SDMA levels.

There are several limitations of this study. Patients without known AF underwent an ECG in clinical routine. It is still possible that AF remained unrevealed in some patients. For this study, we aimed for real world evidence as our patients received standardized stroke diagnostics used in everyday clinical practice. Consequently, not every patient underwent long-term ECG or loop recorder implantation. Nevertheless, we interestingly obtained similar results regarding the biomarker diagnostics as the FIND-AF trial.

## Conclusion

Arg and SDMA can be useful for detection of AF and prediction of AFDAS. However, the incremental value beyond the established clinical risk factors remains modest. The results underscore the previously described biological role of Arg and SDMA in cardioembolic stroke etiology. Further studies should therefore investigate Arg derivatives as potential targets for stroke prevention focusing on endothelial health.

## Supplementary Information

Below is the link to the electronic supplementary material.


Supplementary Material 1


## Data Availability

The datasets used and/or analysed during the current study are available from the corresponding author on reasonable request.

## Non-standard Abbreviations and Acronyms

ADMA: asymmetric dimethylarginine
AF: atrial fibrillation
AFDAS: atrial fibrillation detected after stroke
Arg: L-arginine
AUC: area under the curve
CES: cardioembolic stroke
CI: confidence interval
eCRF: electronic case report form
ESRS: Essen Stroke Risk Score
ESUS: embolic stroke of undetermined source
hArg: homoarginine
KAF: known atrial fibrillation
LAA: large-artery atherosclerosis
LAVI: left atrial volume index
LVEF: left ventricular ejection fraction
mRS: modified Rankin Scale
NIHSS: National Institutes of Health Stroke Scale
NO: nitric oxide
ROC: Receiver operating characteristic
SDMA: symmetric dimethylarginine
SVD: small-vessel disease
TOAST: Trial of Org 10172 in Acute Stroke Treatment

## References

[1] GBD 2021 Stroke Risk Factor Collaborators. Global, regional, and national burden of stroke and its risk factors, 1990–2021: a systematic analysis for the Global Burden of Disease Study 2021. Lancet Neurol. 2024;23(10). 10.1016/S1474-4422(24)00369-7. PMC12254192 39304265

[2] Lin B, Zhang Z, Mei Y, Wang C, Xu H, Liu L, Wang W. Cumulative risk of stroke recurrence over the last 10 years: a systematic review and meta-analysis. Neurol Sci: Off J Italian Neurol Soc Italian Soc Clin Neurophysiol. 2021;42(1). 10.1007/s10072-020-04797-5. 33040195

[3] Ornello R, Degan D, Tiseo C, Di Carmine C, Perciballi L, Pistoia F, Carolei A, Sacco S. Distribution and temporal trends from 1993 to 2015 of ischemic stroke subtypes: a systematic review and meta-analysis. Stroke. 2018;49(4). 10.1161/STROKEAHA.117.020031. 29535272

[4] Hart RG, Diener H, Coutts SB, Easton JD, Granger CB, O’Donnell MJ, Sacco RL, Connolly SJ. Embolic strokes of undetermined source: the case for a new clinical construct. Lancet Neurol. 2014;13(4). 10.1016/S1474-4422(13)70310-7. 24646875

[5] Diener HC, Sacco RL, Easton JD, Granger CB, Bernstein RA, Uchiyama S, Kreuzer J, Cronin L, Cotton D, Grauer C, et al. Dabigatran for prevention of stroke after embolic stroke of undetermined source. N Engl J Med. 2019;380(20). 10.1056/NEJMoa1813959. 31091372

[6] Hart RG, Sharma M, Mundl H, Kasner SE, Bangdiwala SI, Berkowitz SD, Swaminathan B, Lavados P, Wang Y, Wang Y, et al. Rivaroxaban for stroke prevention after embolic stroke of undetermined source. N Engl J Med. 2018;378(23). 10.1056/NEJMoa1802686. 29766772

[7] Geisler T, Keller T, Martus P, Poli K, Serna-Higuita LM, Schreieck J, Gawaz M, Tünnerhoff J, Bombach P, Nägele T et al. Apixaban versus aspirin for embolic stroke of undetermined source. NEJM Evid 2023;3(1). 10.1056/EVIDoa2300235 38320511

[8] Kamel H, Longstreth WT, Jr, Tirschwell DL, Kronmal RA, Marshall RS, Broderick JP, Aragón García R, Plummer P, Sabagha N, Pauls Q, et al. Apixaban to prevent recurrence after cryptogenic stroke in patients with atrial cardiopathy: the ARCADIA randomized clinical trial. JAMA. 2024;331(7). 10.1001/jama.2023.27188. PMC10851142 38324415

[9] Grosse GM, Sieweke JT, Biber S, Ziegler NL, Gabriel MM, Schuppner R, Worthmann H, Bavendiek U, Weissenborn K. Nonstenotic carotid plaque in embolic stroke of undetermined source: interplay of arterial and atrial disease. Stroke. 2020;51(12). 10.1161/STROKEAHA.120.030537. 33040704

[10] Jochmann SL, Sievering EMW, Ernst J, Ringlstetter R, Grosshennig A, Weissenborn K, Grosse GM. Sex-specific risk factors of nonstenotic carotid plaque in embolic stroke of unknown source: a case-control study. Stroke. 2024;55(2). 10.1161/STROKEAHA.123.044833. 38152961

[11] Sieweke J, Biber S, Weissenborn K, Heuschmann PU, Akin M, Zauner F, Gabriel MM, Schuppner R, Berliner D, Bauersachs J, et al. Septal total atrial conduction time for prediction of atrial fibrillation in embolic stroke of unknown source: a pilot study. Clin Res Cardiol. 2020;109(2). 10.1007/s00392-019-01501-2. PMC6989646 31236691

[12] Grosse GM, Biber S, Sieweke J, Martens-Lobenhoffer J, Gabriel MM, Putzer A, Hasse I, van Gemmeren T, Schuppner R, Worthmann H, et al. Plasma dimethylarginine levels and carotid intima–media thickness are related to atrial fibrillation in patients with embolic stroke. Int J Mol Sci. 2019;20(3). 10.3390/ijms20030730. PMC6387438 30744089

[13] Grosse GM, Schwedhelm E, Worthmann H, Choe C. Arginine derivatives in cerebrovascular diseases: mechanisms and clinical implications. Int J Mol Sci. 2020;21(5). 10.3390/ijms21051798. PMC7084464 32150996

[14] Qin Z, Tang L, Huang Q, Chen Y, Zhong W, Tang X. A systematic review of the correlation between serum asymmetric dimethylarginine, carotid atherosclerosis and ischaemic stroke. Eur J Clin Invest. 2021;51(8). 10.1111/eci.13558. 33756002

[15] Riddell A, Flynn A, Bergugnat H, Dowsett L, Miller A. SDMA as a marker and mediator in cerebrovascular disease. Clin Sci (Lond). 2024;138(20). 10.1042/CS20241021. PMC11479986 39391895

[16] Emrich IE, Zawada AM, Martens-Lobenhoffer J, Fliser D, Wagenpfeil S, Heine GH, Bode-Böger SM. Symmetric dimethylarginine (SDMA) outperforms asymmetric dimethylarginine (ADMA) and other methylarginines as predictor of renal and cardiovascular outcome in non-dialysis chronic kidney disease. Clin Res Cardiol. 2018;107(3). 10.1007/s00392-017-1172-4. 29101459

[17] Ziegler NL, Sieweke J, Biber S, Gabriel MM, Schuppner R, Worthmann H, Martens-Lobenhoffer J, Lichtinghagen R, Bode-Böger SM, Bavendiek U, et al. Markers of endothelial pathology to support detection of atrial fibrillation in embolic stroke of undetermined source. Sci Rep. 2019;9(1). 10.1038/s41598-019-55943-9. PMC6923420 31857660

[18] von Elm E, Altman DG, Egger M, Pocock SJ, Gøtzsche PC, Vandenbroucke JP. The strengthening the reporting of observational studies in epidemiology (STROBE) statement: guidelines for reporting observational studies. Int J Surg (London England). 2014;12(12). 10.1016/j.ijsu.2014.07.013.

[19] Adams HP, Bendixen BH, Kappelle LJ, Biller J, Love BB, Gordon DL, Marsh EE. Classification of subtype of acute ischemic stroke. Definitions for use in a multicenter clinical trial. TOAST. Trial of org 10172 in acute stroke treatment. Stroke. 1993;24(1). 10.1161/01.str.24.1.35. 7678184

[20] Joglar JA, Chung MK, Armbruster AL, Benjamin EJ, Chyou JY, Cronin EM, Deswal A, Eckhardt LL, Goldberger ZD, Gopinathannair R, et al. 2023 ACC/AHA/ACCP/HRS Guideline for the diagnosis and management of atrial fibrillation: a report of the american college of cardiology/american heart association joint committee on clinical practice guidelines. J Am Coll Cardiol. 2024;83(1). 10.1016/j.jacc.2023.08.017. PMC11104284 38043043

[21] Uphaus T, Weber-Krüger M, Grond M, Toenges G, Jahn-Eimermacher A, Jauss M, Kirchhof P, Wachter R, Gröschel K. Development and validation of a score to detect paroxysmal atrial fibrillation after stroke. Neurology. 2019;92(2). 10.1212/WNL.0000000000006727. 30530796

[22] Ntaios G, Perlepe K, Lambrou D, Sirimarco G, Strambo D, Eskandari A, Karagkiozi E, Vemmou A, Koroboki E, Manios E, et al. External performance of the HAVOC score for the prediction of new incident atrial fibrillation. Stroke. 2020;51(2). 10.1161/STROKEAHA.119.027990. 31826729

[23] Weimar C, Diener HC, Alberts MJ, Steg PG, Bhatt DL, Wilson PW, Mas JL, Röther J. The Essen stroke risk score predicts recurrent cardiovascular events: a validation within the REduction of Atherothrombosis for Continued Health (REACH) registry. Stroke. 2009;40(2). 10.1161/STROKEAHA.108.521419. 19023098

[24] Martens-Lobenhoffer J, Bode-Böger SM. Quantification of l-arginine, asymmetric dimethylarginine and symmetric dimethylarginine in human plasma: a step improvement in precision by stable isotope dilution mass spectrometry. J Chromatogr B. 2012;904. 10.1016/j.jchromb.2012.07.021. 22884474

[25] Kleindorfer DO, Towfighi A, Chaturvedi S, Cockroft KM, Gutierrez J, Lombardi-Hill D, Kamel H, Kernan WN, Kittner SJ, Leira EC, et al. 2021 Guideline for the prevention of stroke in patients with stroke and transient ischemic attack: a guideline from the American Heart Association/American Stroke Association. Stroke. 2021;52(7). 10.1161/STR.0000000000000375. 34024117

[26] Ghannam M, Al-Qudah AM, Alshaer QN, Kronmal R, Ntaios G, Childs CA, Longstreth WT, Alsawareah A, Keller T, Serna-Higuita LM, et al. Anticoagulation vs antiplatelets across subgroups of embolic stroke of undetermined source: a meta-analysis of randomized controlled trials. Neurology. 2024;103(9). 10.1212/WNL.0000000000209949. PMC12747887 39365971

[27] Michaud GF, Stevenson WG. Atrial fibrillation. N Engl J Med. 2021;384(4). 10.1056/NEJMcp2023658. 33503344

[28] Masood S, Ashraf SMK, Malik MA, Wahab S. P-wave indices and left atrial mechanics as predictors of atrial cardiopathy in embolic stroke of undetermined source. Sci Rep. 2023;13(1). 10.1038/s41598-023-44285-2. PMC10651911 37968274

[29] Sposato LA, Field TS, Schnabel RB, Wachter R, Andrade JG, Hill MD. Towards a new classification of atrial fibrillation detected after a stroke or a transient ischaemic attack. Lancet Neurol. 2024;23(1). 10.1016/S1474-4422(23)00326-5. 37839436

[30] Sposato LA, Chaturvedi S, Hsieh CY, Morillo CA, Kamel H. Atrial fibrillation detected after stroke and transient ischemic attack: a novel clinical concept challenging current views. Stroke. 2022;53(3). 10.1161/STROKEAHA.121.034777. 34986652

[31] Yaghi S, Kamel H, Elkind MSV. Atrial cardiopathy: a mechanism of cryptogenic stroke. Expert Rev Cardiovasc Ther. 2017;15(8). 10.1080/14779072.2017.1355238. PMC6092961 28718666

[32] Hannemann J, Wasser K, Mileva Y, Kleinsang F, Schubert M, Schwedhelm E, Guan K, Wachter R, Böger R. Symmetric dimethylarginine predicts previously undetected atrial fibrillation in patients with ischemic stroke. J Am Heart Association. 2024;13(17). 10.1161/JAHA.124.034994. PMC11646540 39190577

[33] Horowitz JD, De Caterina R, Heresztyn T, Alexander JH, Andersson U, Lopes RD, Steg PG, Hylek EM, Mohan P, Hanna M, et al. Asymmetric and symmetric dimethylarginine predict outcomes in patients with atrial fibrillation: an ARISTOTLE substudy. J Am Coll Cardiol. 2018;72(7). 10.1016/j.jacc.2018.05.058. 30092948

[34] Cordts K, Grzybowski R, Lezius S, Lüneburg N, Atzler D, Neu A, Hornig S, Böger RH, Gerloff C, Magnus T, et al. Guanidino compound ratios are associated with stroke etiology, internal carotid artery stenosis and CHA2DS2-VASc score in three cross-sectional studies. J Neurol Sci. 2019;397. 10.1016/j.jns.2018.12.037. 30640152

[35] Atzler D, Gore MO, Ayers CR, Choe CU, Böger RH, de Lemos JA, McGuire DK, Schwedhelm E. Homoarginine and cardiovascular outcome in the population-based Dallas heart study. Arterioscler Thromb Vasc Biol*.* 2014;34(11). 10.1161/ATVBAHA.114.304398 25189571

[36] Choe C, Lezius S, Cordts K, Gerloff C, Böger RH, Schwedhelm E, Grant PJ. Low homoarginine/SDMA ratio is associated with poor short- and long-term outcome after stroke in two prospective studies. Neurol Sci. 2020;41(1). 10.1007/s10072-019-04058-0. 31482247

[37] Hanff E, Kayacelebi AA, Yanchev GR, Maassen N, Haghikia A, Tsikas D. Simultaneous stable-isotope dilution GC–MS measurement of homoarginine, guanidinoacetate and their common precursor arginine in plasma and their interrelationships in healthy and diseased humans. Amino Acids. 2016;48(3). 10.1007/s00726-015-2120-0. 26573540

[38] Atzler D, Schönhoff M, Cordts K, Ortland I, Hoppe J, Hummel FC, Gerloff C, Jaehde U, Jagodzinski A, Böger RH, et al. Oral supplementation with L-homoarginine in young volunteers. Br J Clin Pharmacol. 2016;82(6). 10.1111/bcp.13068. PMC5099544 27434056

