# Supplementary material for: Symmetric Dimethylarginine for Risk Stratification of Cardioembolic Stroke
Source: Transl Stroke Res. 2026 Jul 14;17(4):79. doi: 10.1007/s12975-026-01460-7 (PMC13368846; doi:10.1007/s12975-026-01460-7)
Supplement: Supplementary file 1 — Supplementary Material 1 [file 12975_2026_1460_MOESM1_ESM.docx]

**Supplementary information**


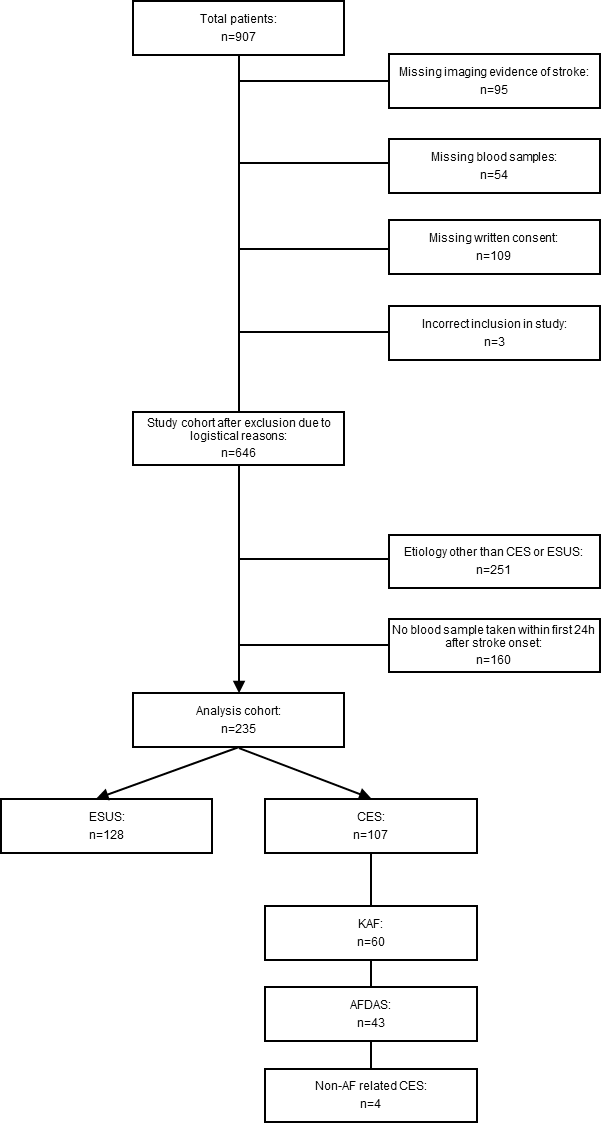


**Fig. S-1:** Study flow chart

**Descriptive comparison of AFDAS and ESUS regarding serum levels and AF-risk scores**

We additionally compared the subgroup of patients with atrial fibrillation detected after stroke (AFDAS) with the embolic stroke of undetermined source (ESUS) collective and found the following:

As shown in Figure S-2, serum levels of Arg were significantly lower in AFDAS patients (median: 100.03 µmol/l, Q1-Q3: 86.00-110.50 µmol/l) compared to ESUS (median: 115.30µmol/l, Q1-Q3: 97.43-133.48 µmol/l). SDMA levels were substantially higher in patients with AFDAS (median: 0.75 µmol/l, Q1-Q3: 0.63-1.02 µmol/l) than in ESUS (median: 0.68 µmol/l, Q1-Q3: 0.60-0.79 µmol/l). There was no substantial difference regarding levels of ADMA in AFDAS (median: 0.58 µmol/l, Q1-Q3: 0.51-0.68 µmol/l) and ESUS (median: 0.59 µmol/l, Q1-Q3: 0.53-0.65 µmol/l).


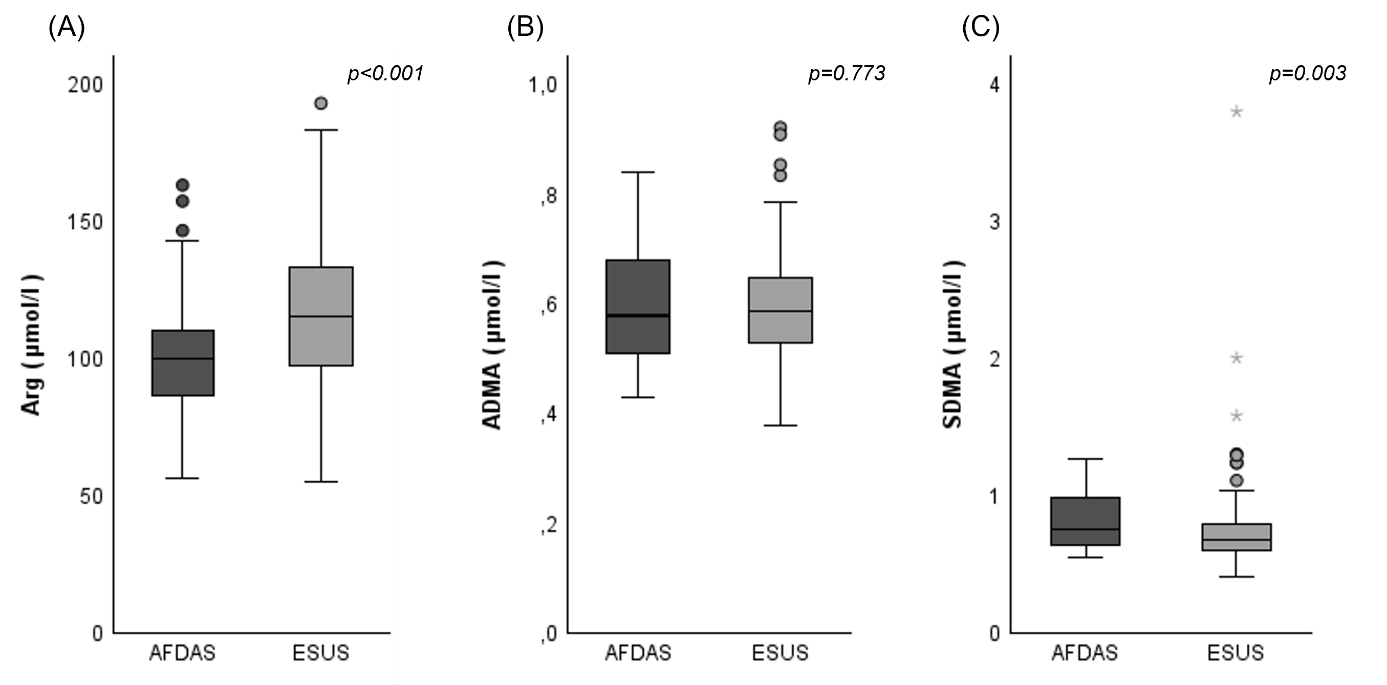


**Fig. S-2:** Comparison of serum concentrations of (A) Arg, (B) ADMA and (C) SDMA between AFDAS and ESUS.

The ratio of Arg/ADMA was substantially lower in patients with AFDAS (median: 166.30, Q1-Q3: 138.64-202.80) compared to ESUS (median: 198.73, Q1-Q3: 170.38-223.85). Arg/SDMA was also lower in patients with AFDAS (median: 130.28, Q1-Q3: 92.06-156.56) than with ESUS (median: 165.53, Q1-Q3: 130.49-209.00). ADMA/SDMA was significantly higher for ESUS (median: 0.86, Q1-Q3: 0.73-0.97) than for AFDAS (median: 0.76, Q1-Q3: 0.63-0.85). (see Figure S-3)


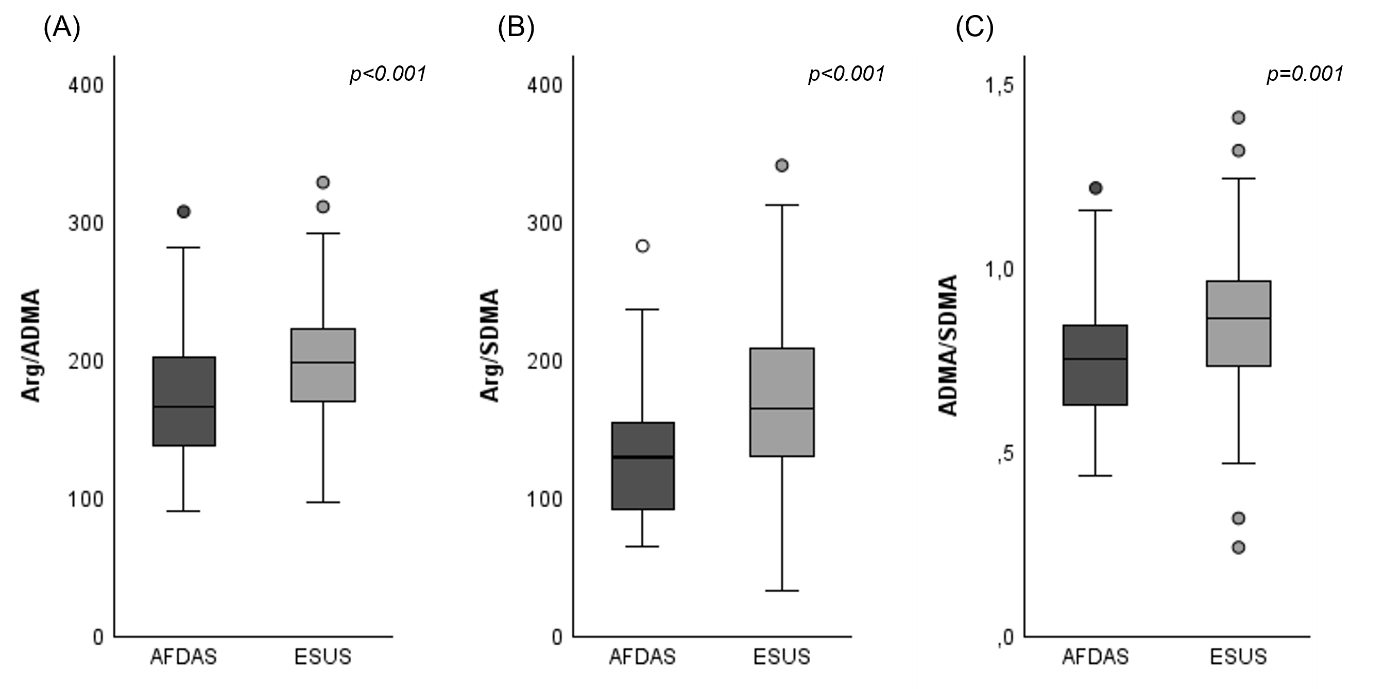


**Fig. S-3:** Comparison of biomarker ratios (Arg/ADMA, Arg/SDMA, ADMA/SDMA) between AFDAS and ESUS: (A) Arg/ADMA, (B) Arg/SDMA and (C) ADMA/SDMA

**Binary regression and ROC analysis to distinguish between AFDAS and ESUS**

To differentiate between AFDAS (n=43) and ESUS (n=128) the analysis revealed an OR per increase= 1.05 (95%CI: 0.03-35.01, p=0.979) with an AUC of 0.52 (95%CI: 0.41-0.62, p=0.103) regarding ADMA-levels, an OR per increase= 1.03 (95%CI: 1.01-1.04 p< 0.001) with an AUC of 0.68 (95% CI: 0.59-0.77, p<0.001) for Arg and an OR per increase= 0.56 (95%CI: 0.20-1.54, p= 0.259) with an AUC of 0.65 (95% CI: 0.56-0.74, p=0.001) for SDMA. The optimal SDMA cutoff to discriminate AFDAS from ESUS was 0.61 µmol/l (sensitivity: 0.93, specificity: 0.33).

The analysis for biomarker ratios revealed an OR per increase= 1.015 (95%CI: 1.007 – 1.023, p< 0.001) with an AUC of 0.72 (95% CI: 0.63-0.81, p<0.001) for Arg/SDMA, an OR per increase= 1.015 (95%CI: 1.006-1.024, p = 0.002) with an AUC of 0.68 (95% CI: 0.58-0.78, p<0.001) for Arg/ADMA and an OR per increase = 17.32 (95%CI: 2.39-125.51, p = 0.005) with an AUC of 0.67 (95% CI: 0.57-0.76, p<0.001) for ADMA/SDMA.

Among AF risk scores, the AS5F score had an OR per increase=0.91 (95%CI: 0.87.0.95, p<0.001) with an AUC of 0.76 (95% CI: 0.67-0.84, p<0.001), while for HAVOC the OR per increase was 0.80 (95%CI: 0.67-0.95, p=0.011) with an AUC of 0.67 (95% CI: 0.58-0.75, p<0.001), for CHA₂DS₂-VASc the OR per increase was 0.70 (95%CI: 0.53-0.93, p=0.015) with an AUC of 0.62 (95%CI: 0.53-0.70, p=0.007) and for ESRS the OR per increase was 0.84 (95%CI: 0.65-1.09, p=0.200) with an AUC of 0.58 (95%CI: 0.49-0.66, p=0.081) (see Figure S-4). The incremental value of Arg/SDMA ratio added to the clinical score AS5F is only modest in AFDAS- vs. ESUS patients (Delta-AUC = 0.02 (95%CI: -0.07-0.1) (see Figure S-5).


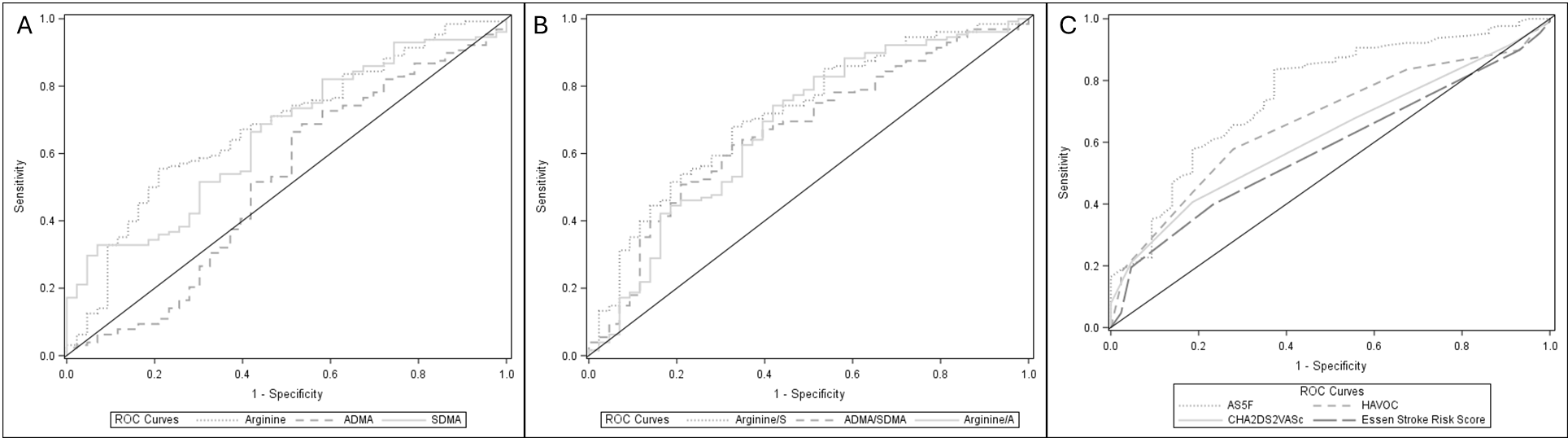


**Fig. S-4:** ROC-analysis for distinguishing between AFDAS and ESUS with (A) biomarker levels, (B) biomarker ratios and (C) AF-scores.


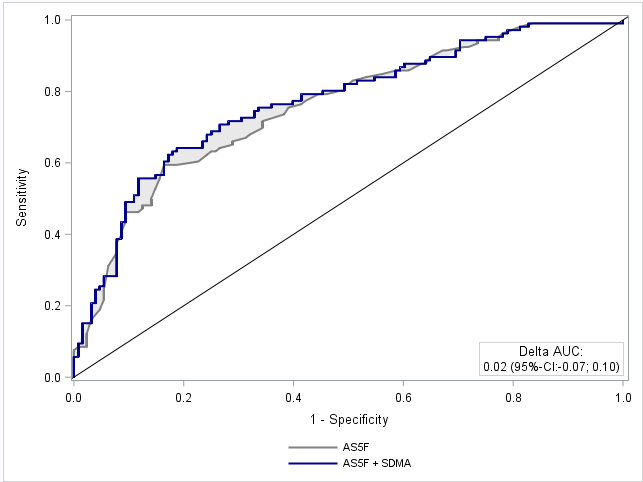


**Fig. S-5:** Delta-AUC comparing AS5F-score combined with Arg/SDMA ratio to isolated AS5F-score in patients with AFDAS vs. ESUS.
